# Application of Co-Culture Technology to Enhance Protease Production by Two Halophilic Bacteria, *Marinirhabdus* sp. and *Marinobacter hydrocarbonoclasticus*

**DOI:** 10.3390/molecules26113141

**Published:** 2021-05-24

**Authors:** Hoang Thi Hong Anh, Esmaeil Shahsavari, Nathan J. Bott, Andrew S. Ball

**Affiliations:** School of Science, RMIT University, Bundoora, Melbourne, VIC 3083, Australia; e.shahsavari@gmail.com (E.S.); nathan.bott@rmit.edu.au (N.J.B.); andy.ball@rmit.edu.au (A.S.B.)

**Keywords:** co-culture, enzyme production, halo-bacteria, protease activity, salinity

## Abstract

Although axenic microbial cultures form the basis of many large successful industrial biotechnologies, the production of single commercial microbial strains for use in large environmental biotechnologies such as wastewater treatment has proved less successful. This study aimed to evaluate the potential of the co-culture of two halophilic bacteria, *Marinirhabdus* sp. and *Marinobacter hydrocarbonoclasticus* for enhanced protease activity. The co-culture was significantly more productive than monoculture (1.6–2.0 times more growth), with *Marinobacter hydrocarbonoclasticus* being predominant (64%). In terms of protease activity, enhanced total activity (1.8–2.4 times) was observed in the co-culture. Importantly, protease activity in the co-culture was found to remain active over a much broader range of environmental conditions (temperature 25 °C to 60 °C, pH 4–12, and 10–30% salinity, respectively). This study confirms that the co-culturing of halophilic bacteria represents an economical approach as it resulted in both increased biomass and protease production, the latter which showed activity over arange of environmental conditions.

## 1. Introduction

In biotechnology, there have been numerous, spectacular examples where axenic microbial cultures and their enzymes have developed into a multi-billion dollar industry [1]. For example amylases from *Bacillus subtilis*, glucosidases from *Aspergillus flavus,* proteases from *Aspergillus niger,* and lactases from *Saccharomyces cerevisiae* [2,3,4]. These enzymes are used in various industries (e.g., food, pharmaceutical, textile, paper, leather, and energy) [5]. In these examples, the use of a single microbial strain for enzyme production is successful on the basis that:

1. The substrate used is defined and constant.
2. The conditions of operation of the microbial product are stringent and consistent.
3. The products are of significant commercial value.

When considering the application of a microbial inoculum to enhance the treatment of wastewater from the fish industry it is difficult to develop microbial cultures as many of these criteria are not met [6,7]. Yet, high levels of nitrogen and organic compounds in fish-processing wastewater entering the rivers and oceans have become key factors in the pollution of receiving water, especially in coastal areas [8]. As a result, the fish-processing industry globally is facing increasingly stringent environmental regulations [9]. However, the treatment of wastewater from fish processing is complicated by several factors including high salinity, high concentrations of N and protein, and the variable nature of the waste [10]. Several approaches have been trialed to remove pollutants from fish-processing wastewater [11], including chemical methods such as the addition of FeCl_3_ and copolymers, or physical approaches using sedimentation and dissolved air flotation [12]. However, both physical and chemical treatments have limitations due to the production of secondary compounds and costs associated with the treatment process, largely due to their high energy consumption and the high salt concentration [13]. Biological processes are particularly well suited for wastewater containing high nitrogen concentrations and do not result in secondary pollution or residues [10]. However, wastewater from the fish-processing industry is highly variable, not only in terms of salinity but also in temperature and pH. Salinity varies depending on the specific process undertaken, ranging from 3% to 21% [14,15,16]; the pH of raw effluents from fish processing varies from 5.9 to 6.8 [17,18] while the temperature is dependent on the ambient conditions and processing treatment [19].

In this case, despite an urgent need, the development of a single commercial microbial strain for use in enhancing protein degradation in fish-processing wastewater treatment is unlikely to be widely applicable. Yet in natural microbial communities, the most effective and resilient communities are those that contain microbial strains capable of degrading similar substrates, but over differing environmental conditions (e.g., temperature, pH, and salinity) allowing ecosystems to function under a range of conditions, thereby assisting in the resilience of the ecosystem to environmental change [20,21]. This concept is also adopted in environmental biotechnology. For example, the accumulation of acids in biogas reactors, due to overloading with organic substances, can inhibit methanogenesis by reducing the pH to less than the optimal range for these bacterial groups. However, in a mixed culture consisting of *Methanosarcina mazei* and *Clostridium butyrium, C. butyricum* converts glycerol into 1,3-propanediol as a major product, but also produces significant amounts of acetate, formate, and butyrate as inhibiting by-products. *M. mazei* relieves this inhibition through the utilization of the by-products for energy production [22]. Similar findings were reported for a co-culture of *Escherichia coli* K12 and *Acinetobacter baylyi* ADP1; these two strains exhibited a symbiotic nature in terms of substrate utilization and growth under aerobic conditions. This resulted in a three-fold increase in growth in the co-culture compared to the monoculture; the directed carbon flow resulted in a four-fold increase in acetate removal in the co-culture [23]. 

In previous work, *Marinirhabdus* strain HTHA1 and *Marinobacter hydrocarbonoclasticus* strain HTHA2, two halophilic bacteria isolated from seawater were found to be capable of efficient nutrient removal from fish wastewater with salinity above 3% [24]. Protease production from the independent culture of the two bacteria were 14.31 and 10.12 U·mL^−1^, respectively. As a result of their augmentation into non-sterile fish wastewater, both chemical oxygen demand (COD) and nitrogen removal in the wastewater were significantly increased. Since the costs associated with the use of multiple single cultures which are ultimately combined for use as bioaugmentation agents may limit the commercial success of the application, as well as neglecting the benefit of rationally engineered co-cultures for synthetic biology applications, it is essential to evaluate the economic value of co-culture as a means of enhancing commercial potential. Here the application of co-culture technology for growth and protease production of the two strains in one bioreactor was investigated, followed by an assessment of the stability of the protease activity of the cell-free culture to variations in temperature, salinity, and pH. Two hypotheses were tested: first, both growth and protease activity in co-culture results in greater biomass and protease production compared to monoculture, and second, the range of activity of the protease in terms of pH temperature and salinity will be enhanced in co-culture compared to the individual protease activities from the monocultures. 

## 2. Results

### 2.1. Bacteria Growth and Protease Activity—Comparison of Mono and Co-Cultures

**Figure 1:**
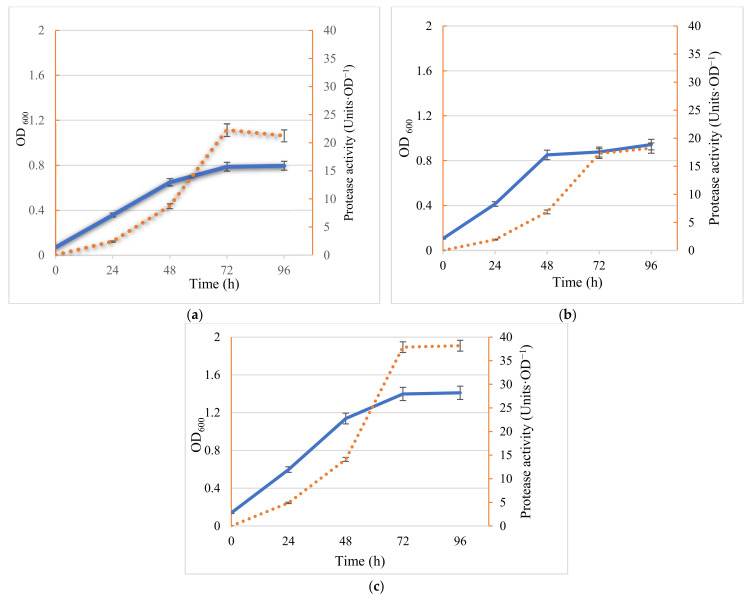
Cell growth (solid lines) was monitored using OD_600_ and extracellular protease activity (dashed lines) was determined using casein as a substrate. (**a**) Inoculated *Marinirhabdus* sp.; (**b**) inoculated *Marinobacter* sp.; (**c**) inoculated co-culture of *Marinirhabdus* sp. and *Marinobacter* sp. Results represent the means of three experiments, and bars indicate ± standard deviation.

Monocultures and mixed cultures of the two bacteria were inoculated into the marine broth and incubated at 37 °C with agitation. Cell growth and extracellular protease activity were monitored throughout the incubation. No significant lag phase was observed and by 48 h, all cultures reached the end of the exponential phase; the stationary phase was reached by 72 h. Importantly, the biomass of mixed cultures (OD_600_ = 1.392) reached more than 2.0 and 1.6 times that of the two pure cultures for *Marinirhabdus* sp. and *Marinobacter* sp. respectively (OD_600_ = 0.700 and OD_600_ = 0.877) (Figure 1).

Along with increased biomass production in the co-culture, protease activity also increased (Figure 1). In fact, not only was there a two-fold increase in biomass in the co-culture, in terms of protease production per unit OD, activity was significantly greater in the co-culture; 38.6 units of protease activity OD^−1^, which was 1.8–2.4 times that of pure cultures (21.6 and 16.0 units of protease activity OD^−1^, for *Marinirhabdus* sp. and *Marinobacter* sp. respectively) (Figure 1). 

Plate counting showed that *Marinobacter* sp. predominated, accounting for 26% to 74% (4.1 × 10^6^ and 11.6 × 10^6^ cells·mL^−1^, respectively) of the colony-forming units present in the co-culture (Table 1). This ratio reflects the results from the monoculture where *Marinobacter* sp. grew markedly better in the marine medium than *Marinihabdus* sp. (Table 1). 

### 2.2. Effect of pH, Temperature, and Salinity on Protease Activity

**Figure 2:**
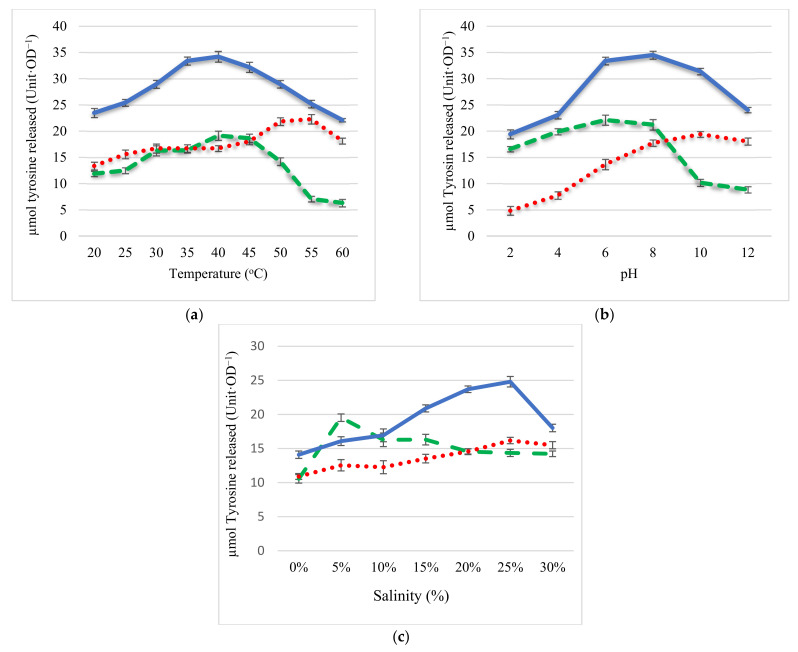
Effect of temperature (**a**), pH (**b**), and salinity (**c**) on *Marinirhabdus* sp., *Marinobacter* sp., and coculture protease activity. Solid line—co-culture; dashed line (----)—*Marinirhabdus* sp.; dotted line (····)—*Marinobacter* sp., pH was determined at 37 °C in different buffers by varying pH values (pH 2–12; temperature was determined by assessing protease activity at different temperatures (20–60 °C); salinity was investigated in the range of 0 to 30% at 37 °C, pH 7.5. Results represent the means of three experiments, and bars indicate ± standard deviation.

The second hypothesis was that the proteases from the two bacteria will, together, in terms of the range of activity provide a much more resilient commercial process than proteases from the monocultures. Here we compared the effects of changes in temperature, pH, and salinity on the activity of proteases from monocultures and co-cultures of *Marinirhabdus* sp. and *Marinobacter* sp. The protease activity of the co-culture was more effective over both temperature and pH ranges than those of monocultures (Figure 2a,b). In terms of temperature, between 30 °C and 45 °C, the activity of the coculture protease was 1.4–1.8 times higher than that of the protease from *Marinirhabdus* sp. and *Marinobacter* sp, respectively. Importantly, protease activity from the co-culture showed approximately 35 (Units·OD^−1^) protease activity over a wide range of temperatures (25–60 °C); protease activity from *Marinirhabdus* sp. showed maximal activity between 30 and 45 °C (18 Units·OD^−1^) while protease activity from *Marinobacter* sp. showed maximal activity (22 Units·OD^−1^) between 45 and 60 °C (Figure 2a). Furthermore, in terms of pH, protease activity from the co-culture remained high over a wide range of pH 4 to 12 (27–30 Units·OD^−1^); in contrast, protease activity from the monocultures showed greater variation at different pH values (Figure 2b). Protease activity from *Marinirhabdus* sp. showed maximum protease activity at pH 6.0 (22 Units·OD^−1^) under acid-neutral conditions (pH 4–8) in contrast, protease activity from *Marinobacter* sp. exhibited the highest activity in an alkaline-neutral environment (pH 8–10), with greatest activity at pH 10 (19 Units·OD^−1^). Results comparing the effect of salinity on protease activity show that protease activity from *Marinirhabdus* sp. was active within a salinity range of 5–15% and maximal at 5% (20 Units·OD^−1^); the optimal salinity for protease activity from *Marinobacter* sp. ranged from 20–30% (protease activity 15–17 Units·OD^−1^) (Figure 2c). Protease activity in the co-culture however, displayed a broader spectrum, from 10% to 30% with protease 1.2–1.7 times higher than that of monocultures (17–25 Units·OD^−1^).

## 3. Discussion

The metabolic activity in terms of the degradation of the various carbon and nitrogen-containing compounds present in fish-processing wastewater are key criteria when considering the application of a microbial inoculum to enhance the removal of COD and TN. In a previous study, two mono halophilic bacteria *Marinihabdus* sp. and *Marinobacter hydrocarbonoclasticus* were isolated and found to be most as bioaugmentation agents in terms of protease production and the enhanced removal of COD and TN from fish-processing wastewater when combined [25]; their addition resulted in the effluent reaching the European Union’s (EU) discharge standard (level B, COD < 120 mg·L^−1^, TN < 70 mg·L^−1^). In this study, co-culturing of the two halophilic bacteria *Marinihabdus* sp. and *Marinobacter hydrocarbonoclasticus* resulted in significantly increased biomass and protease activity compared to monoculture (Figure 1). These results support the hypothesis that the two organisms are capable of enhanced growth in co-culture, although the mechanisms to promote this synergistic growth are yet to be elucidated. Previous work suggested that both intracellular metabolism and cell–cell communication via metabolic cooperation were essential in determining the population dynamics of the ecosystem [26]. Zhou et al. reasoned that the metabolites produced during the co-culture of *B*. *megaterium* and *K. vulgare* in soft agar were increased compared with those in the monocultures due to the population dynamics of the mixed culture [26].

The results confirm that co-cultivation represents a viable approach to biomass and enzyme production with numerous advantages. In co-culture, not only was there a two-fold increase in biomass (OD_600_ values of 1.39 compared with 0.70 and 0.88, respectively for *Marinihabdus* sp. and *Marinobacter hydrocarbonoclasticus*) but protease activity was 1.8–2.4 times greater than that of pure cultures (21.6 and 16.0 Units·OD^−1^, respectively). When comparing the protease activity from halophilic bacteria recorded in the current study with those reported in the literature one study reported that the isolation of three halophilic bacterial, SP1(1), SP1(2b), and SP3(2) cultured from salterns produced protease activity of 18.16, 9.99, and 12.76 Units·OD^−1^, respectively [27]. These findings are similar to the results of two monocultures in this study (21.6 and 16.0 Units·OD^−1^, respectively), but significantly lower in terms of protease activity than the co-culture (38.6 Units·OD^−1^). To the best of our knowledge, this is the first report on the production and characterization of protease isolated from the co-culture of halophilic bacteria. Currently, it is unclear how different species contribute to the consortia, but this study showcases the fact that co-cultivation has numerous advantages. Previously it was shown that an *Escherichia coli* co-culture successfully produced high levels of *cis*-, *trans*-muconic acid, and 4-hydroxybenzoic acid, while exhibiting low-efficiency sugar mixture utilization [28]. In another example, three species, *E. coli, Bacillus subtilis,* and *Shewanella oneidensis* formed a cross-feeding microbial consortium which enhanced the performance of a bio-electrochemical system with potential application in bioenergy production [29]. In both cases, the co-culture system outperformed the monoculture system in direct comparison. The present study showed the specific combination of *Marinirhabdus* sp. and *Marinobacter hydrocarbonoclasticus* may be important, as bacteria in mixed culture promote synergistic growth. In the current study cell-free culture extracts was assessed, rather than preparing purified enzyme. Interestingly when crude enzymes from the co-culture were added to saline fish-processing wastewater, enhanced TN and COD removal was observed [25].

Previously it has been proposed that species in co-culture produce complementary enzymes that participate in metabolite cross-feeding which can result in synergistic growth [30]. Some species produce toxic metabolites, such as lactate which can be used by other consortia members, thus allowing the bacteria to utilize complex substrates in a cooperative manner [31]. Likely, the metabolites produced by one bacteria were used as substrates to increase the microbial biomass of the other bacteria which in turn increased the activities of the enzymes [32]. This represents a significant commercial saving if the same amount of biomass can be produced with co-culture as with two systems with monoculture.

Overall, in this study both hypotheses have been proved correct; first, both growth and protease activity in co-culture contribute to an increased production of biomass and proteases compared to monoculture, and second, the wider range of protease activity in co-cultures compared with individual protease activates from monocultures. Co-culture of the two halophilic protease producing strains significantly enhanced biomass and protease activity leading to reduced production costs. 

In terms of temperature, pH, and salinity, protease activity from the co-culture exhibited greater activity over a broader range than that of monocultures. In co-culture, the optimum temperature was 25 to 60 °C for protease activity. While the protease activity of the co-culture decreased by 35% as the temperature rose from 40 °C to 60 °C, over the same temperature range proteases from *Marinihabdus* sp. and *Marinobacter* sp. reduced by 67% and 40%, respectively. The optimum pH for the protease activity from the co-culture was 4 to 12, while the optimum pH for proteases from *Marinihabdus* and *Marinobacter* was 4 to 8 and 8 to 12, respectively. Protease activity remained high in the salinity range of 10–30% in co-culture, while in individual cultures of *Marinihabdus* sp. and *Marinobacter* sp., protease activity was high only between 5% and 15% and from 20% and 30%, respectively. In comparison to the activity of proteases reported in the literature, the range of activity of the proteases from the co-cultures appears greater. For example, the protease activity from *Bacilus proteolyticus* CFR3001, isolated from fish-processing wastes (both freshwater and marine) showed a specific activity of 22.05 Units·OD^−1^ at 37 °C, and was active between 40 °C and 50 °C but lost >20% of its activity around 60 °C [33]. In other study, the protease activity of a halophilic bacteria, strain ZY8^T^, isolated from a rock salt was optimal at a salinity of 15–20%, pH 7.0–9.0, 20–45 °C [34]. In this study the application of co-culture technology resulted in a wider activity range of protease, and there are no reports where co-culture systems of halophilic bacteria have been used in commercial processes. This work supports the idea of further exploiting co-culturing for the production of bacterial inoculation. Table 2 summarizes the advantages of the co-culture determined in this study.

## 4. Materials and Methods 

### 4.1. Reagents and Equipment

All reagents were of analytical grade and were purchased from Sigma Co. (St Louis, MO, USA).

### 4.2. Bacteria

Seawater (1 L) from Port Philip Bay, Australia (38°9′0″ S, 144°52′0″ E) was collected in a sterile container and used within 4 h of collection. Cultivation and isolation experiments were performed using marine broth media. Seawater (1 mL) was inoculated into marine broth (100 mL, 3% salinity (*w*/*v*)) and incubated for 24 h at 37 °C and 150 rpm [35]. Following incubation, a serial dilution plating method was followed for isolation. Culture (1 mL) was inoculated onto Petri dishes containing marine agar using a spread plate technique. Following inoculation, plates were incubated at 37 °C for 48 h [36]. The isolates were then purified using a standard technique [37]. Selected isolates were inoculated into marine broth (5 mL) under aseptic conditions and incubated at 150 rpm and 37 °C for 72 h.

DNA extraction, PCR amplification, sequencing, and subsequent analysis were used to identify bacteria [24]. The strain HTHA1 was identified as *Marinirhabdus* sp (GenBank accession MG889587), and the strain HTHA2 was identified as *Marinobacter* sp. (99% identical to *Marinobacter hydrocarbonoclasticus* with GenBank registration number MG252259*)*. These two strains were confirmed as halophilic bacteria in a previous study [24].

Subsequent tests demonstrated their efficacy as individual species to reduce both total nitrogen and COD in fish-processing wastewater [24]. Isolates were maintained on marine agar plates (Difco^TM^ Marine agar 2216, Bacto Laboratories PTY LTD, Mount Pritchard, Australia) [38]. Plates were incubated at 37 °C for 3 days and then stored at 4 °C. All culture media were sterilized by autoclaving.

### 4.3. Culture Conditions

A single colony of each bacteria was inoculated in 25 mL of marine broth (Difco^TM^ Marine broth 2216, Bacto Laboratories PTY LTD, Mount Pritchard, Australia) and incubated at 37 °C and 120 rpm for 56 h according to the findings of a previous study using the Taguchi method to optimize the living conditions (unpublished data). For monocultures, an aliquot (0.2 mL) of each prepared culture was inoculated into two different tubes containing 25 mL of marine broth. For co-culture, an aliquot (0.1 mL) of the two bacteria was inoculated in one tube containing 25 mL of marine broth. Tubes were incubated at 37 °C and 120 rpm. Bacterial growth was monitored by measuring absorbance at 600 nm in a spectrophotometer (SPECTROstar Omega; BMG LABTECH, Offenburg, Germany). All experiments were performed in triplicate.

To investigate which bacteria were dominant in the co-culture, a plate count method [39] was used, made possible by the different phenotypes of the colonies from the two organisms. An aliquot of mixed culture (1 mL) after 56 h of incubation was serially diluted and plated on marine agar and incubated for 48 h at 37 °C. Following growth, colonies were counted on plates containing between 30 and 300 colonies.

### 4.4. Enzyme Preparations

To test whether the mixed inoculum resulted in enhanced protease activity, following 56 h incubation in marine broth, mono- and co-cultures were centrifugated at 4 °C at 10,000× *g* for 10 min. Supernatants were used to compare the protease activity between mixed and pure cultures modified as previously described [40]. Supernatants (20 µL) were added to casein (100 µL, 0.65% (*w*/*v*)) in 50 mM potassium phosphate buffer (pH 7.5). The mixture was incubated for 10 min at 37 °C. The reaction was terminated by adding 10% trichloroacetic acid (80 µL); the mixture was kept for 30 min at room temperature and then centrifuged for 15 min at 10,000× *g*. Filtrate (30 µL)-mixed with 500 mM sodium carbonate solution (150 µL) and Folin & Ciocalteu’s Phenol Reagent (20 µL) was immediately added. When the protease in the samples digested casein, the amino acids tyrosine was liberated and free tyrosine reacts with Reagent to produce a blue colored chromophore, which was measured for an absorbance value on the spectrophotometer at 660 nm (SPECTROstar Omega; BMG LABTECH, Offenburg, Germany). One unit of enzyme activity was defined as the amount of enzyme required to liberate 1 µmol of tyrosine per min under the defined assay conditions. A range of (50–250 µg·mL^−1^) tyrosine concentration was used for the construction of a standard calibration curve.

### 4.5. Effect of pH, Temperature, and Salinity on Protease Activity in Mono and Mixed Culture 

After 56 h of incubation at 37 °C in marine broth, mono- and mixed bacteria cultures were centrifugated at 4 °C and the supernatants were used to investigate the effect of pH, temperature, and salinity on protease activity. The effect of pH, temperature, and salinity on protease activity was assayed by varying one parameter at a time while keeping other conditions constant. The different conditions included: (i) different pH conditions ranging from 2 to 10 with a constant temperature of 37 °C and salinity 3%, pH modifications (pH 2.0–12.0) were achieved by replacing phosphate buffer in the reaction mixture with Briton and Robinson Universal buffer [41]; (ii) varying temperature from 20 °C to 60 °C at a constant incubation pH 7.5 and salinity 3%; (iii) varying salinity from 0% to 30% with a constant incubation temperature 37 °C, pH 7.5. Control experiments were also performed in which supernatants were held in an ice-bath for 5 min following heat treatment and before determining enzyme activity. All experiments were performed in triplicate.

### 4.6. Statistical Methods

Data obtained from the study were analyzed using the SPSS statistical package (version 26–IBM, New York, NY, USA). Data were subjected to analyses of variance (ANOVA), and means were compared by Duncan’s test. Statistical significance was determined at *p* < 0.05 level when the F value was significant. Data were represented as mean ± standard deviation (SD) of the three replicates. 

## 5. Conclusions

The aims of this study were (i) to determine whether the growth and protease activity in co-culture of two halophilic bacteria results in greater biomass production and protease yields compared to monoculture; and (ii) to assess the range of activity of the protease enhanced in co-culture compared to the individual protease activity from the monocultures. The results of the present study confirmed that co-culture resulted in enhanced growth and protease activity. The mixed culture reached higher densities than pure cultures (1.6–2.0 times) as well as exhibiting enhanced protease activity (1.8–2.4 times). Additionally, protease activity in the co-culture showed a broader optimum pH (from 4 to 12), temperature (25 °C–60 °C), and salinity (10–30%). The overall conclusion of this study is that microbial consortia can be effectively grown, with the resultant product (in this case proteases) being more robust in terms of efficacy over a range of environmental conditions. This enhances the commercial viability of this environmentally friendly bioaugmentation; however, more studies are required to better assess the effect of coculture in terms of protease activity on improving industrial wastewater treatment such as aquaculture effluents.

## Figures and Tables

**Table 1:** Characteristics of recognizably distinct colony types on marine agar plates and relative abundance of the two bacterial strains in the co-culture.

Bacteria	Image of the Colony	Distinguishing Characteristics
*Marinihabdus* sp.	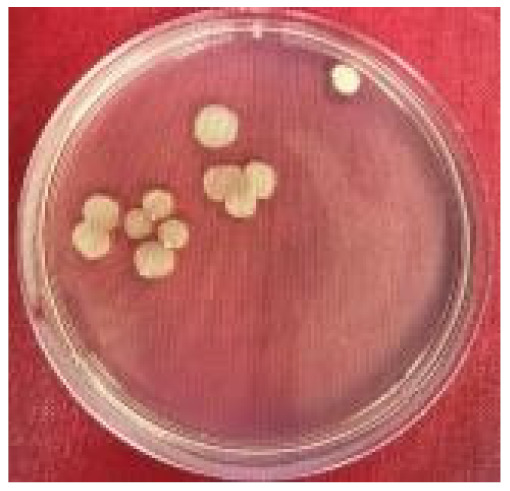	Circular shape; moderate and large size (>3 mm in diameter). Shiny and smooth surface; Light yellow color and the margin is entire.
*Marinobacter* sp.	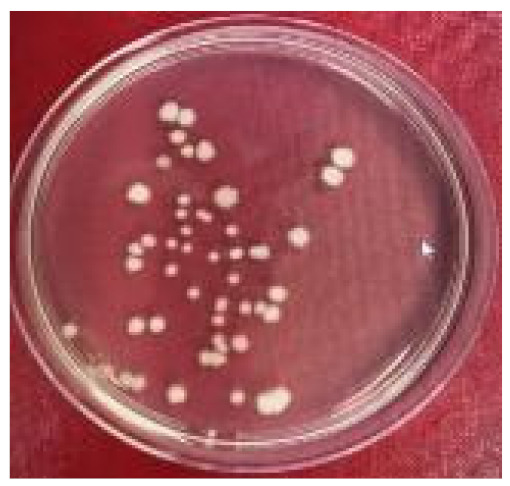	Circular shape; small and medium size (1–3 mm in diameter). Opaque and rough surface; milk-white color, and the margin is curled.
Number of colonies of *Marinihabdus* sp. and *Marinobacter* sp. (cells·mL^−1^) in co-culture at dilution 10^−5^	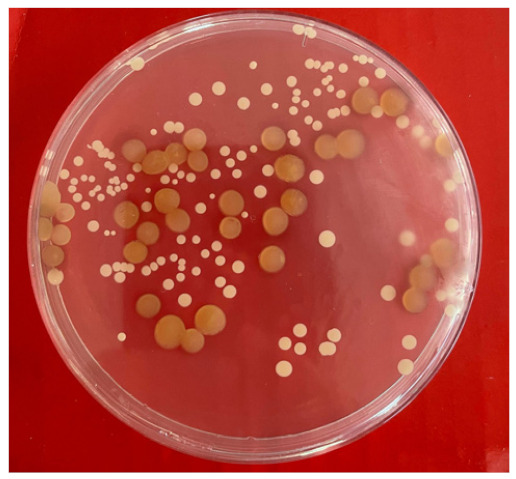	*Marinihabdus* sp.*:* 4.1 × 10^6^ (cells·mL^−1^) *Marinobacter* sp.*:* 11.6 × 10^6^ (cells·mL^−1^)

**Table 2:** The efficacy of coculture compared to monoculture.

Sample	Biomass (OD_600_)	Effect of Temperature on Protease Activity		Effect of pH on Protease Activity		Effect of Salinity on Protease Activity	
		Optimum Temperature * (t^o^)	Protease Activity (Units·OD^−1^)	Optimum Ph *	Protease Activity (Units OD^−1^)	Optimum Salinity * (%)	Protease Activity (Units·OD^−1^)
*Marinihabdus*	0.788	30–45	6.3–19.2	4–8	8.8–21.2	5–15	10.5–19.5
*Marinobacter*	0.960	45–60	13.4–22.3	8–12	4.8–19.4	20–30	10.9–16.1
Co-culture	1.398	25–60	22.1–34.2	4–12	19.4–34.5	10–30	14.0–24.7

* Optimum temperature, pH, and salinity range were defined as the range of conditions in which 75% of the maximum activity was observed for each sample.

## Sample Availability

Samples of the compounds are not available from the authors.
